# Efficacy of Leuprorelin 3-Month Depot (11.25 mg) Compared to 1-Month Depot (3.75 mg) for Central Precocious Puberty in Chinese Girls: A Prospective Cohort Study

**DOI:** 10.1155/2022/1043293

**Published:** 2022-12-22

**Authors:** Jianmei Yang, Qijun Song, Shuo Gao, Yuye Gao, Xiaohong Shang, Guimei Li, Yan Sun, Xiaoping Luo

**Affiliations:** ^1^Department of Pediatric Endocrinology, Shandong Provincial Hospital Affiliated to Shandong First Medical University, Jinan 250021, Shandong, China; ^2^Department of Pediatric Endocrinology, Shandong Provincial Hospital, Cheeloo College of Medicine, Shandong University, Jinan 250021, Shandong, China; ^3^State Key Laboratory of Generic Manufacture Technology of Chinese Traditional Medicine, Lunan Pharmaceutical Group Co., Ltd., Linyi 276006, Shandong, China; ^4^Shandong First Medical University, Jinan 250000, Shandong, China; ^5^Department of Pediatrics, Tongji Hospital, Tongji Medical College, Huazhong University of Science and Technology, Wuhan 430030, China

## Abstract

**Objective:**

A 3-month depot of leuprorelin acetate (LA) was introduced in China in July 2020. However, the clinical experience is limited. The purpose of this study was to compare the efficacy of a LA 11.25 mg 3-month depot with that of a 3.75 mg 1-month depot in suppressing pubertal development for the treatment of central precocious puberty (CPP). *Subjects and Methods*. A prospective study, including 78 girls with CPP treated with LA, was conducted. 31 patients were treated with a LA 3-month depot, and 47 were treated with a LA 1-month depot. Participants were interviewed at baseline and 6 months. Anthropometric, metabolic, and reproductive data were assessed at each interview. Bone age, serum endocrine hormones, maximum diameter of uterus and volume of ovary of each patient were evaluated. A pharmacoeconomic evaluation was also conducted.

**Results:**

Treatment with a 3-month depot was similar to treatment with a 1-month depot in terms of baseline characteristics. After 6 months of treatment, a suppressed level of luteinizing hormone (LH) (LH < 2.5 IU/L) was found in 100% and 95% of the 11.25 mg and 3.75 mg groups, respectively. LH decreased from 2.11 ± 1.83 and 2.82 ± 2.31 at baseline (*P*=0.172) to 0.37 ± 0.39 and 0.44 ± 0.76 (*P*=0.758) in the 3-month and 1-month groups, respectively. Follicle stimulating hormone and estradiol levels, bone age/chronological age (BA/CA), height velocity, maximum diameter of uterus and volume of ovary did not show any distinction between the two groups after 6 months of treatment, but both were significantly ameliorated compared with the baseline. The loss of working time of parents and study time of patients and the numbers of visits, injections and laboratory examinations obviously decreased in the 3-month depots.

**Conclusion:**

An LA 3-month depot was equally effective and safe as a 1-month depot for hormonal suppression and bone maturation inhibition, providing clinical experience in China. The 11.25 mg depot of LA is a safe, efficient, and economical treatment method for the advanced activation of the hypothalamic-pituitary-gonadal (HPG) axis.

## 1. Introduction

Through the activation of the hypothalamic-pituitary-gonadal (HPG) axis, gonadal stimulation leads to an increase in sex steroid secretion, resulting in the early onset of secondary sexual traits and is related to a sudden increase in growth and accelerated skeletal maturation that affects adult height [1]. Central precocious puberty (CPP) is a common disease caused by premature activation of the HPG axis and results in the onset of puberty before 8 years of age in girls and 9 years of age in boys [2]. The principal aim of CPP treatment is to suppress gonadotrophin secretion and the associated secretion of gonadal sex steroids [3]. Gonadotropin-releasing hormone analogues (GnRHas) are the recommended treatment for activation of the HPG axis. These drugs inhibit gonadotropin secretion by desensitizing and downregulating GnRH receptors by releasing GnRH in a steady rather than pulsatile manner, resulting in the decline of gonadal steroids to the level of prepubertals [1, 4]. Analogues of GnRH reverse or stabilize pubertal development, thereby resuming puberty and increasing height without affecting puberty resumption [5, 6].

GnRHas are widely used in the treatment of CPP [1, 7]. There are no obvious adverse effects following therapy, and the drug is released gradually, improving short-andlong-term outcomes for patients [1, 8, 9]. In short-term studies, it was shown that the 3-month depot formulation of GnRHa was effective in patients [10–12]. However, there has rarely been a comparison of the effectiveness of 3.75 mg versus 11.25 mg depot in suppressing the HPG axis [12–14]. In many countries of the world, leuprorelin acetate (LA) has been used in patients suffering from CPP in depot formulations that are injected monthly or every 3 months [7, 13]. The use of the 3-month formulation, given that the therapy might take several years, is expected to improve patient adherence and assure better quality of life. Thus, to support the clinical administration of the 11.25 mg depot to treat CPP in China, more data are needed.

This study compared the security, efficacy, and compliance of LA 3-month depot treatment and LA 1-month depot treatment in Chinese girls with CPP.

## 2. Patients and Methods

Our patients were recruited for a pilot prospective cohort study during the period from July 2019 to December 2021 at the Shandong Provincial Hospital Affiliated with Shandong First Medical University. This study was performed in accordance with the ethical standards laid down in the Declaration of Helsinki and the Medical Ethics Committee of Shandong Provincial Hospital affiliated with Shandong First Medical University (Ethics approval code is SWYX: NO.2020-147). Patients provided written informed consent before laboratory and clinical examinations, and parent/guardian consent was obtained for patients under the age of 16.

The following criteria were used to determine CPP: development of the breasts before 8 years of age, peak level of luteinizing hormone (LH) > 5.0 IU/L, and LH peak/follicle stimulating hormone (FSH) peak ratio >0.6 on a GnRH stimulation test (2.5 *µ*g/kg i.v.) [15, 16]. Additionally, we included the appearance of breast buds between the ages of 8 and 10 years, accompanied by the presence of pubic or axillary hair and/or accelerated growth rate or bone age greater than 2 SD above chronological age [17].

The following criteria were used for exclusion: (1) Patients with missing vital data; (2) Boys with CPP; (3) Girls with CPP whose cause can be identified, such as brain tumours; (4) Patients with chronic diseases, such as epilepsy and asthma; (5) Patients with any organic diseases, such as hypothyroidism. Ultimately, 78 girls were selected and enrolled in the present study.

### 2.1. Anthropometric Measurements and Laboratory Methods

Among 78 patients, 31 received an LA 3-month depot, and 47 received an LA 1-month depot. LA 3-month depots were given every three months, whereas LA 1-month depots were conducted monthly. Both were given by intramuscular injection. All patients who received LA 3-month depots weighed over 20 kg. Participants were interviewed regularly, and data was collected at baseline and 6-month treatments. Anthropometric, metabolic, and reproductive parameters were assessed at each interview. At the beginning of treatment, the age, weight (kg), BMI and tanner stage were evaluated, and peak values were collected after GnRha stimulation. The height (cm), bone age (BA), BA/chronological age (CA), serum LH, FSH and estradiol (E2) levels, maximum diameter of uterus (U-MD), volume of left ovary (LO-V) and right ovary (RO-V) of every patient were analysed at the start of therapy, as well as half a year after treatment. BMI is calculated as weight in kilograms divided by height in metres squared (kg/m^2^). Height velocity was calculated. Pubertal development is based on the Tanner stage, and BA is based on the Greulich-Pyle method. Bone age advancement was determined by BA/CA. Serum LH, FSH, and E2 concentrations were measured by electrochemiluminescence immunoassay methods (Roche, Basel, Switzerland). The lipid profile and fasting plasma glucose (FPG) levels were measured by a biochemistry analyser (Olympus, Japan) for calibration. Following triptorelin-stimulated measurement, we determined the serum peak levels of LH and FSH. Additionally, the levels of fasting serum LH < 2.5 IU/L after therapy were considered to suppress the HPG axis [14].

### 2.2. Evaluation of Economic Psychology

A survey of patients who received injections of LA was conducted to estimate the fee for GnRHa, loss of working time (parents) and studying time (CPP girls), pain and anxiety due to injections, numbers of visits and examinations. To evaluate economic psychology, the respondents were asked to rate their degree of pain and anxiety. For example, possible answers to the question “how much pain did you feel due to injection?” included: no pain-0, mild pain-1, moderate pain-4, severe pain-7 For the question “how much anxiety did you feel due to injection?” possible answers included: no anxiety-0, mild anxiety-1, moderate anxiety-4, severe anxiety-7.

### 2.3. Statistical Analysis

All statistical analyses were performed using SPSS version 18.0 for Windows (Chicago, IL, USA). Values are expressed as the mean ± standard deviation (SD). Student's *t*-tests were used to compare groups. Categorical variables were analysed using the *χ*^2^ test instead. *P* < 0.05 was deemed statistically significant in all tests.

## 3. Results

The baseline characteristics of patients at the beginning of LA treatment, including age, FPG, lipid profile, height, weight, BMI, bone age, pubertal stage, LH and FSH (basal and peak) levels, E2 levels, and ovary volumes, are shown in Table 1. There were no significant differences in auxological and hormonal features between the two groups (Table 1). The levels of peak LH and FSH (GnRH-stimulated) were no statistically significant difference between the two groups.

### 3.1. Hormonal and Gonadal Suppression

**Figure 1:**
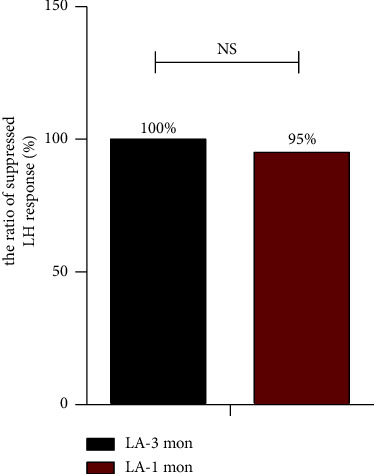
Suppressed LH response after treatment with LA 3-month and LA 1-month depots. Data are presented as number/total number (%). LH < 2.5 IU/L indicates suppressed LH response. LA = Leuprorelin acetate, NS=No significance.

The LA 3-month group had 14/14 (100%) responders with an adequately suppressed LH response after 6 months, and the LA 1-month group had 35/37 (95%) responders. After 6 months, there were no significant differences in the proportion of responders between the two groups (Figure 1). There were no significant differences in serum LH, FSH and E2 levels at half a year of therapy between the two groups. Furthermore, the changes over time of LH, FSH and E2 (∆LH, ∆FSH and ∆E2) were also no significant differences between the two groups. The above results indicate that hormone suppression did not show significant differences between the two groups after treatment (Table 2).

There were no significant differences in the maximum diameter of the uterus (U-MD), the volume of the left ovary (LO-V) and the volume of the right ovary (RO-V) after 6 months of treatment between the two groups. Additionally, the changes over time of U-MD, LO-V and RO-V (∆U-MD, ∆LO-V and ∆RO-V) were also no significant differences between the two groups. These results indicate that the gonadal suppression did not show significant differences between the two groups after treatment (Table 3).

### 3.2. Clinical Efficacy

Both groups were compared to their baseline after 6 months of treatment. The Tanner stage was 2 or 3 and evenly distributed when the therapy began in both groups. After 6 months, neither the LA 3-month group nor the LA 1-month group showed progress in breast development. As a result of treatment, the height velocity was 9.16 ± 3.2 cm/year for the LA 3-month group and 7.42 ± 1.43 cm/year for the LA 1-month group. The two groups did not differ significantly (Table 4).

There were no significant differences between the two groups in terms of bone age advancement (Table 4). BA/CA decreased from 1.26 ± 0.13 and 1.21 ± 0.13 years at baseline (*P*=0.138) to 1.22 ± 0.10 and 1.18 ± 0.12 years after half a year in girls using 11.25 mg and 3.75 mg, respectively (*P*=0.001). Furthermore, ∆BA/∆CA was no significant difference between the two groups. These results indicate that both treatments were clinically effective (Table 4).

### 3.3. Evaluation of Economic Psychology

Between the two groups, there was no significant difference in fee for GnRHa, pain and anxiety due to injections, number of gynaecological ultrasounds and BA examinations. Compared to LA 1-month, the loss of working time of parents and study time of girls and the numbers of visits, injections and laboratory examinations at half a year decreased obviously in the LA 3-month depot (Table 5). Therefore, the 3-month LA had the advantages of convenience and safety, improving compliance and medical efficiency.

## 4. Discussion

GnRHa are commonly used to treat HPG axis activation because of their safety and efficacy [1]. To date, the most popular choice for treatment has been the depot GnRHa given for one month. However, over the past ten years, a depot offering a three-month dose has become available. The 1-month depot containing 3.75 mg of LA is incorporated into a matrix of a poly (glycolic acid, lactic acid) copolymer at a ratio of 1 : 3 [18], the vehicle used for the newly developed LA 3-month (11.25 mg) depot contains only polylactic acid [19]. Several studies have demonstrated the efficacy of the 3-month depot, but most of them had few sample quantities or limited long-termfollow-up and/or comparative data are available [9, 20]. The LA 3-month (11.25 mg) depot is the first long-actingsustained-release drug approved in China. As far as we known, there are no study comparing the effect of 11.25 mg and 3.75 mg depots on the HPG axis in China. We observed the efficacy of LA 3-month compared to LA 1-month depots in suppressing gonadotropin secretion in girls with CPP in China. Our results showed that LA 3-month depots were similarly effective to LA 1-month depots. Although our study is a single center, short-term study, we have seen the report of Chung et al. [14] from Chorea, which is larger and have a longer follow-up period. They found that a suppressed levels of LH was seen in 93.5% and 100% of the girls treated with the 3-month and 1-month depots after 1 year of treatment, respectively (*P*=0.226). Height velocity showed no significant difference between the two groups. Degree of bone age advancement decreased from 1.22 ± 0.07 and 1.22 ± 0.08 years at baseline (*P*=0.914) to 1.16 ± 0.07 and 1.17 ± 0.08 in the girls treated with the 3-month and 1-month depots after 1 year, respectively (*P*=0.481). Their study showed that the efficacy of long-acting triptorelin 3-month was comparable to 1-month depot regarding hormonal suppression and inhibition of bone maturation. The triptorelin 11.25 mg 3-month depot is an effective treatment for girls with CPP. It is consistent with our research results.

The criteria for the biochemical efficacy of adequate LH suppression during GnRHa therapy are controversial. Lucaccioni et al. using the measurement of urinary gonadotropins for assessment and management of pubertal disorder [21]. An evaluation of HPG suppression also can be conducted by examining serum LH or sex steroid in unstimulated or stimulated (following GnRHa administration) [22–25]. There is still controversy over the appropriate LH suppression cut-off value with GnRHa stimulation [26–28]. In our study, we used LH < 2.5 IU/L as the cut-off value for LH suppression, which is consistent with previous studies [14]. There was no significant difference in suppression of LH between the two groups, and serum LH and serum E2 concentrations declined approximately 7-10-fold from baseline to 6 months after treatment. We found no differences in HPG axis suppression between the 3.75-mg 1-month and 11.25-mg 3-month doses. Because the application time of 3-month LA in China is limited, many parents have doubts about choosing this dosage with uncertainty of validity. The above results could provide a good explanation for the effectiveness of 3-month LA.

Furthermore, we observed the efficacy of the LA 3-month depot in terms of height velocity and bone age advancement. In both groups, height velocity dropped after 6 months of treatment, without a significant difference between them, which was consistent with the study of Fuld et al. [12]. In both groups, BA/CA was significantly reduced after half a year of therapy, and there was no significant difference in the two groups, indicating adequate clinical efficacy in both treatments. Suppressed bone maturity of the LA 3-month depot was also reported in a long-term study of a 2-year injection [12].

Our study could give many Chinese families the confidence to choose LA 3-month depots. In the present study, it was demonstrated that the LA 3-month depot is very effective in suppressing the progression of the HPG axis, which was similar to the LA 1-month depot. There were few adverse reactions to LA 3-month, such as subcutaneous induration, local inflammation, and allergy. The adverse reactions were mild and curable, and no one stopped the drug because of the adverse reactions. The LA 3-month treatment showed similar safety profiles to the 1-month depot without new safety questiones Additionally, some advantages of LA 3-month are recognized, such as a reduced number of doctor visits and injections, elimination of transportation and accommodation costs, amelioration of the doctor's outpatient burden and even avoidance of the risk of hospital visits during the COVID-19 epidemic. Therefore, the LA 3-month depot had the advantages of efficacy, safety, convenience, and improved compliance and medical efficiency.

This study had several limitations. First, it was a single-center study. Second, long-term studies are necessary to measure effects of an LA 3-month depot until the reproductive axis has recovered. Since boys with CPP are few and difficult to balance in the two groups, we need to gather more data on boys in future studies.

## 5. Conclusion

The long-acting LA 3-month depot to be as effective as the 1-month depot for hormonal inhibition and bone maturation suppression. The LA 3-month depot is an effective, safe and economical treatment for suppressing the HPG axis. We suggest that both the LA 11.25 mg dosage form and the LA 3.75 mg dosage form can be widely used to manage CPP in China.

## Figures and Tables

**Table 1:** Baseline characteristics of the LA 3-month depot and 1-month depot groups.

Variables	LA-3 mon (*n* = 31)	LA-1 mon (*n* = 47)	*P* value
Age (yr)	8.89 ± 0.80	9.19 ± 1.05	0.312
FPG (mmol/L)	5.40 ± 0.42	5.35 ± 0.49	0.779
Serum TC (mmol/L)	4.22 ± 0.42	4.57 ± 0.91	0.370
Serum TG (mmol/L)	1.10 ± 0.27	1.40 ± 0.72	0.302
Serum LDL-C (mmol/L)	2.38 ± 0.15	2.57 ± 0.61	0.440
THt (cm)	159.43 ± 3.78	160.08 ± 3.29	0.450
Height (cm)	139.32 ± 5.56	140.55 ± 6.62	0.401
Weight (kg)	34.68 ± 5.14	37.05 ± 9.34	0.205
BMI (kg/m^2^)	17.85 ± 2.24	18.54 ± 3.49	0.337
Tanner stage			
2	22/31 (70.97%)	30/46 (65.22%)	
3	9/31 (29.03%)	16/46 (34.78%)	
Bone age (yr)	10.96 ± 0.59	10.84 ± 0.94	0.527
Serum basal LH (IU/L)	2.11 ± 1.83	2.82 ± 2.31	0.172
Serum basal FSH (IU/L)	4.11 ± 1.31	4.65 ± 1.68	0.155
Serum peak LH (IU/L)	27.91 ± 19.16	27.29 ± 17.94	0.901
Serum peak FSH (IU/L)	13.17 ± 4.89	14.35 ± 8.53	0.524
Serum peak LH/FSH	6.75 ± 4.15	5.64 ± 4.59	0.545
Serum E2 (pg/ml)	24.44 ± 11.84	29.39 ± 15.35	0.205
U-MD (cm)	2.48 ± 0.48	2.69 ± 0.58	0.141
RO-V (ml)	2.50 ± 0.93	3.07 ± 1.45	0.101
LO-V (ml)	2.44 ± 0.95	2.98 ± 1.28	0.106

Data are presented as mean ± standard deviation or number (%). Abbreviations: LA, Leuprorelin acetate; FPG, fasting plasma glucose; TC, total cholesterol; TG, triglycerides; LDL-C, low-density lipoprotein cholesterol; THt, target height; BMI, body mass index; LH, luteinizing hormone; FSH, follicle stimulating hormone; E2, estradiol; U-MD, maximum diameter of uterus; LO-V, volume of left ovary; RO-V, volume of right ovary.

**Table 2:** The serum LH, FSH and E2 levels after leuprorelin acetate injection.

Group	LH (mIU/mL)			∆LH (mIU/mL)	FSH (mIU/mL)			∆FSH (mIU/mL)	E2 (pg/mL)			∆E2 (pg/mL)
	Baseline	6-mon	*P* value		Baseline	6-mon	*P* value		Baseline	6-mon	*P* value	
LA3-mon	2.11 ± 1.83	0.37 ± 0.39	0.002	1.89 ± 1.79	4.11 ± 1.31	1.15 ± 0.66	0.001	3.12 ± 1.34	24.44 ± 11.84	2.50 ± 1.00	0.001	20.95 ± 12.18
LA1-mon	2.82 ± 2.31	0.44 ± 0.76	0.001	1.84 ± 2.20	4.65 ± 1.68	1.01 ± 0.85	0.001	3.62 ± 2.08	29.39 ± 15.35	3.84 ± 3.77	0.001	24.97 ± 11.98
*t*	1.381	0.309		−0.079	1.437	0.262		0.819	1.284	1.317		0.840
*P* value	0.172	0.758		0.938	0.155	0.592		0.418	0.205	0.194		0.408

Data are presented as mean ± standard deviation. Abbreviations: LH, luteinizing hormone; FSH, follicle-stimulating hormone; E2, estradiol; LA, leuprorelin acetate.

**Table 3:** The U-MD, LO-V and RO-V after leuprorelin acetate injection.

Group	U-MD (cm)			∆U-MD (cm)	LO-V (mL)			∆LO-V (mL)	RO-V (mL)			∆RO-V (mL)
	Baseline	6-mon	*P* value		Baseline	6-mon	*P* value		Baseline	6-mon	*P* value	
LA3-mon	2.48 ± 0.48	2.45 ± 0.35	0.192	0.18 ± 0.36	2.44 ± 0.95	1.53 ± 1.04	0.048	0.73 ± 1.08	2.50 ± 0.93	1.68 ± 0.74	0.048	0.79 ± 1.17
LA1-mon	2.69 ± 0.58	2.53 ± 0.54	0.077	0.23 ± 0.59	2.98 ± 1.28	1.78 ± 1.12	0.024	1.19 ± 1.28	3.07 ± 1.45	2.01 ± 0.89	0.031	0.90 ± 1.26
*t*	1.494	0.544		−0.269	1.651	0.691		0.863	1.669	1.232		0.207
*P* value	0.141	0.590		0.790	0.106	0.494		0.400	0.101	0.226		0.838

Data are presented as mean ± standard deviation. Abbreviations: U-MD, maximum diameter of uterus; LO-V, volume of left ovary; RO-V, volume of right ovary.

**Table 4:** Changes of bone age and height velocity in girls after treatments with LA.

Variables	LA3-mon	LA1-mon	*P* value
BA/CA			
0 mon	1.26 ± 0.13	1.21 ± 0.13	0.138
6 mon	1.22 ± 0.10	1.18 ± 0.12	0.290
*t*	6.528	3.992	
*P* value	0.001	0.001	
∆BA/∆CA	0.67 ± 0.73	0.69 ± 0.89	0.920
Height velocity (cm/year)	9.16 ± 3.2	7.42 ± 1.43	0.137

Data are presented as mean ± standard deviation (number). Abbreviations: LA, leuprorelin acetate; BA, bone age; CA, chronological age.

**Table 5:** Evaluation of economic psychology for the LA 3-month depot and 1-month depot in half a year.

Variables	LA-3 mon	LA-1 mon	*P* value
Fee for GnRHa, CNY	7820.63 ± 257.59	7681.33 ± 303.80	0.079
Loss of working time (day)	2.33 ± 1.56	4.14 ± 2.29	0.001
Loss of studying time (day)	1.48 ± 0.96	2.52 ± 1.77	0.008
^a^Pain due to injections, score	2.56 ± 1.74	2.62 ± 1.91	0.912
^a^anxiety due to injections, score	1.63 ± 1.62	1.38 ± 1.56	0.589
Times of visits	1.97 ± 0.31	5.86 ± 0.66	0.001
Number of injections	1.97 ± 0.31	5.86 ± 0.91	0.001
Times of gynaecological ultrasound	1.28 ± 0.58	1.57 ± 0.60	0.085
Times of BA	1.28 ± 0.58	1.29 ± 0.56	0.978
Times of laboratory examination	1.91 ± 0.59	2.52 ± 1.21	0.016

^a^The pain/anxiety degrees: total score is 10, no (score 0), mild (score 1), moderate (score 4), severe (score 7). Abbreviations: CNY, Chinese Yuan.

## Data Availability

The data used to support the findings of this study can be obtained from the corresponding author upon request.
